# Inverse relationship between Epstein-Barr virus serostatus and anti-glutamic acid decarboxylase autoantibody levels

**DOI:** 10.1007/s12026-026-09778-y

**Published:** 2026-04-18

**Authors:** Cyril Debuysschere, Mahdi Ouafi, Magloire Pandoua Nekoua, Delphine Lobert, Pascal Pigny, Enagnon Kazali Alidjinou, Didier Hober

**Affiliations:** 1 Laboratoire de virologie ULR3610, Univ Lille et CHU Lille, Lille, France; 2 Service Hormonologie Métabolisme Nutrition Oncologie, CHU de Lille, Lille, France

**Keywords:** Epstein Barr virus, EBV, Autoimmunity, Pancreatic islet autoimmunity, Autoantibodies, Glutamic acid decarboxylase, GAD antibodies

## Abstract

Epstein-Barr virus (EBV) is suspected of being involved in the pathogenesis of several autoimmune diseases. However, its role in pancreatic islet autoimmunity remains to be determined. In the present study, the relationship between EBV serostatus and serum level of glutamic acid decarboxylase autoantibodies (GADA), a common marker of pancreatic islet autoimmunity, was investigated. Serum samples were collected from 158 individuals (aged 2–25 years) with GADA levels ≥ 0.7 U/mL and the EBV serostatus of each individual was determined. EBV seropositive (EBV+) individuals were defined as positive for both serum VCA IgG and EBNA IgG and EBV seronegative (EBV-) individuals were negative for these antibodies. Univariate and multivariate analyses were performed to investigate the association between EBV serostatus and GADA levels. EBV+ individuals tend to have lower GADA levels than EBV- individuals (*p* = 0.053). After adjustment for gender and age, mean GADA levels were significantly lower in EBV+ individuals than in EBV- individuals (9.34 U/mL versus 17.49 U/mL, respectively, *p* = 0.015). The lower GADA levels in EBV+ individuals suggest that there is an inverse relationship between pancreatic islet autoimmunity and EBV serostatus. Further studies are needed to investigate the role of EBV in pancreatic islet autoimmunity.

## Introduction

Epstein-Barr virus (EBV) is a ubiquitous human enveloped DNA virus of the *Orthoherpesviridae* family within the *Gammaherpesvirinae* subfamily. It infects naïve B cells and epithelial cells and establishes lifelong latency in the host by persisting as an episome in memory B cells, with periodic reactivation [1]. EBV is suspected of being involved in the pathogenesis of several autoimmune diseases (AID) including multiple sclerosis (MS) [2], systemic lupus erythematosus (SLE), among others [3]. In type 1 diabetes (T1D), the EBV-specific humoral immune response has been studied [4, 5]. Immunoproteomic analysis revealed an antibody response to a wider range of EBV epitopes in T1D patients compared to controls, suggesting a relationship between EBV infection and islet autoimmunity [5].

In T1D, individuals with genetic susceptibility may develop autoantibodies specific to pancreatic islets. The autoantibodies most commonly used in clinical practice to assess pancreatic islet autoimmunity are anti-glutamic acid decarboxylase 65 (GAD), anti-tyrosine phosphatase-like protein (IA2), anti-insulin (IAA), anti-zinc transporter 8 (ZnT8) and anti-islet cell (ICA), which are measurable in serum samples [6]. Among these, anti-GAD antibodies (GADA) are widely used due to their high prevalence in both pediatric and adult patients with islet autoimmunity and because they appear years before the clinical onset of T1D [7]. Moreover, GADA remain the most prevalent positive autoantibodies for many years after T1D diagnosis and β-cell destruction [8].

The issue of the relationship between EBV and autoimmunity directed against pancreatic islets was addressed by studying EBV serostatus and serum levels of anti-GAD autoantibodies (GADA), a common marker of pancreatic islet autoimmunity.

## Methods

### Population selection

All serum samples received and analyzed at CHU de Lille (Lille, France) between 1 January 2024 and 30 April 2025 with GADA levels ≥ 0.7 U/mL (Limit of detection), measured once (intra-assay CV: 10.0% for low values (~ 0.8 U/mL) and 1.3% for high values (~ 45.9 U/mL) (CentAK^®^ anti-GAD_65_ M radioimmunoassay, Medipan, Germany). The samples were selected from the routine analysis biobank. To focus on individuals with pancreatic islet autoimmunity, we excluded patients with GADA-related neurological disorders, suspected type 2 or gestational diabetes, other autoimmune diseases, individuals over 25 years of age, islet transplant recipients, and samples originating from gynecology or neurology departments.

### EBV serostatus

VCA IgG and EBNA IgG antibodies were measured in serum samples by chemiluminescence using Abbott VCA IgG and EBNA-1 IgG kits on the Alinity immunoassay instrument (Abbott Laboratories, USA). Individuals positive for both antibodies were classified as EBV seropositive (EBV+), those negative for both as seronegative (EBV−), and those with only one antibody were excluded.

### Ethical considerations

This research project complies with the MR004 reference methodology of the French National Commission on Informatics and Liberty (CNIL) and has been registered in the institution’s data processing registry under the number DEC24-265.

### Analysis of results

Statistical analyses and graphics were performed using XLSTAT 2021.5 (Addinsoft, Paris, France) and GraphPad Prism v8.0.2 (GraphPad Software, San Diego, CA). Age and GADA levels were firstly compared between EBV + and EBV- groups using Mann-Whitney U tests due to non-normal distributions (Shapiro-Wilk). Sex ratio and EBV seropositivity were compared using a Chi² test. Results are shown as median and interquartile range [Q1-Q3]. The association between age, sex, and EBV seropositivity was examined using multivariable logistic regression. Results were expressed as odds ratio (OR) and 95% confidence interval (95% CI). Multiple linear regression evaluated the independent associations of age, sex, and EBV status with GADA levels, and in EBV+ individuals, the associations of VCA IgG and EBNA IgG with GADA levels. Because GADA values showed right-skewness, a log10 transformation was applied before regression. Coefficients were back-transformed (10^β^) and expressed as ratio of geometric means and 95% CI.

## Results

A total of 158 serum samples with GADA levels ≥ 0.7 U/mL were obtained from individuals aged 2–25 years. EBV serostatus of these individuals was assessed by measuring VCA IgG and EBNA IgG in their serum. Both antibodies were detected in 82 individuals. Sixteen participants showed atypical profiles (12 with isolated VCA IgG and 4 with isolated EBNA IgG) and were excluded.

The final cohort included 142 individuals: 82 EBV+ (57.75%) and 60 EBV- (42.25%). The median age was 14 years [9–18], the sex ratio was 79 males (55.63%) for 63 females (44.37%), and median GADA level was 14.75 U/mL [2.98–55.88], ranging from 0.7 to 89.7 U/mL.

Sex ratio did not differ significantly between EBV+ (53.33% males) and EBV- individuals (57.31% males) (*p* = 0.733). EBV+ individuals were older than EBV- individuals (15 years [10–20] vs. 12.5 years [7–15], *p* = 0.001). In the multivariable model, each additional year of age increased the odds of EBV seropositivity by 11% (OR = 1.11; 95% CI: 1.04–1.12; *p* = 0.001), with no effect of gender (OR = 1.04; 95% CI: 0.52–2.09; *p* = 0.915).

**Fig. 1:**
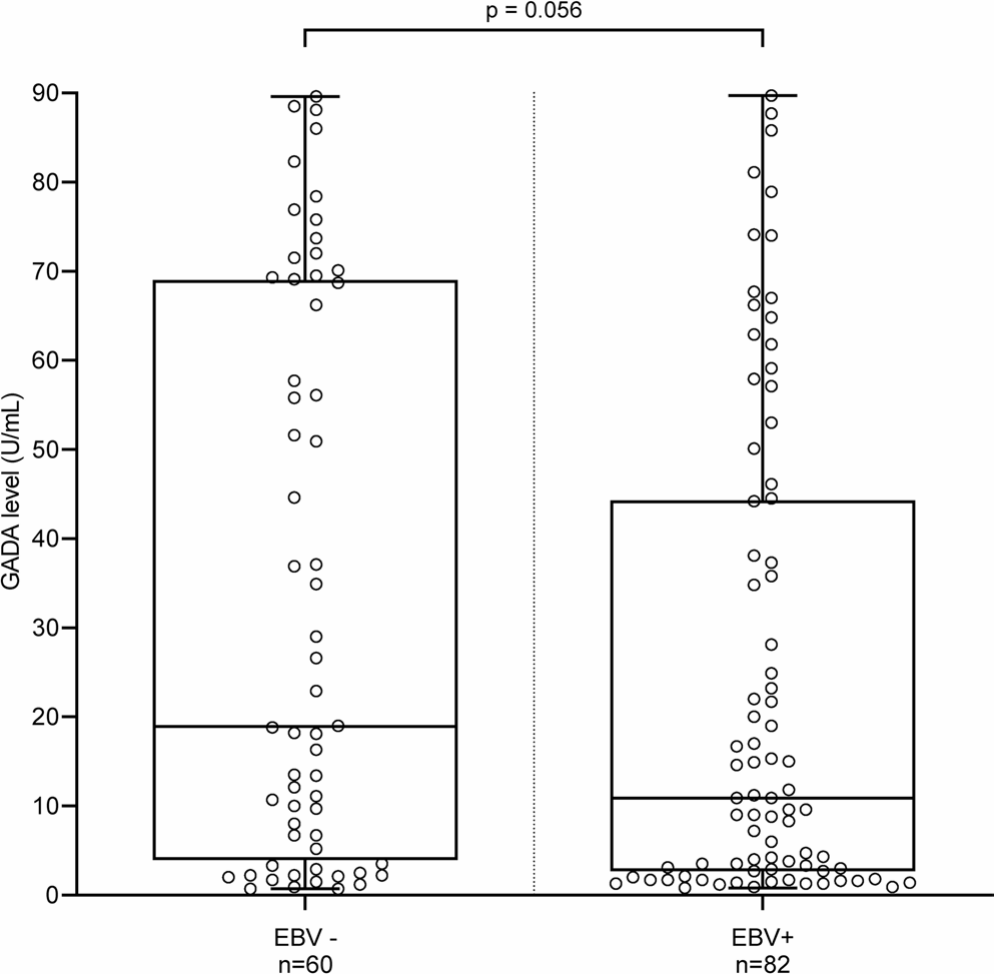
Serum GADA levels according to EBV serostatus. Individual values are represented, boxplots show the median (horizontal line), the first and third quartiles (box bounds), and the minimum and maximum values (whiskers). *P* value: Mann-Whitney U test (univariate analysis).

GADA levels were lower in EBV+ individuals (10.90 U/mL [2.7-44.28]) compared with EBV− individuals (18.90 U/mL [3.93-69]) in the univariate analysis, although the difference did not reach statistical significance (*p* = 0.053) (Fig. 1). As EBV+ individuals were older than EBV− individuals, and in order to assess the independent effect of EBV seropositivity, age and sex on GADA levels, a multiple linear regression was performed. Each additional year of age was associated with a 6% higher mean GADA level (ratio = 1.06, 95% CI: 1.02–1.11, *p* = 0.005).

**Fig. 2:**
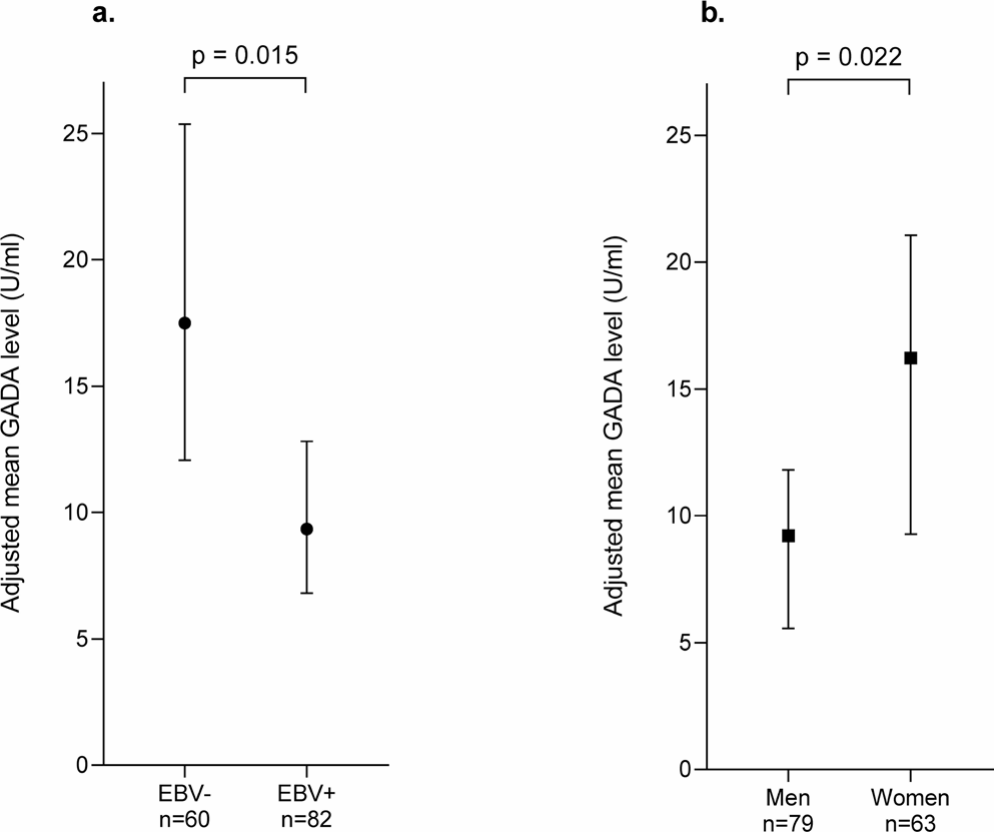
Adjusted mean serum GADA levels, by EBV status (**a**) and by sex (**b**). Multivariable analyses adjusting for age, sex, and EBV serostatus were conducted to assess serum GADA levels. Graphs represent adjusted mean GADA levels (U/mL) with 95% confidence intervals according to EBV serostatus (**a**) and according to sex (**b**)

Multivariate analysis found that EBV+ individuals have 46% lower mean GADA levels than EBV- individuals, adjusted mean of 9.34 U/ml vs. 17.49 U/ml respectively (ratio = 0.535; 95% CI: 0.32–0.88, *p* = 0.015) (Fig. 2a). Moreover, men have approximately 43% lower mean GADA levels than women, adjusted mean 9.21 U/ml vs. 16.22 U/ml respectively (ratio = 0.57; 95% CI: 0.35–0.92, *p* = 0.022) (Fig. 2b). The regression model was significant (F = 5.175, *p* = 0.002) and explained 10.1% of the variance (R² = 0.101).

Multiple linear regression found no influence of VCA IgG or EBNA IgG levels on log-transformed GADA levels in EBV+ individuals (*p* = 0.642 and *p* = 0.517, respectively).

## Discussion

A relationship between EBV and AID is suspected. It has been suggested that the virus could serve as a necessary trigger or catalyzer in autoimmunity, particularly in MS or SLE [9, 10], but the precise role of the virus in the pathogenesis of these diseases remains unclear. Although genetic predisposition is a key factor, studies on migrants and siblings, as well as the increasing incidence of T1D in recent years, suggest that environmental factors play a critical role in pancreatic islet autoimmunity [11]. EBV has been linked to T1D in several studies, with evidence of a distinct humoral response to EBV antigens in individuals with T1D [4, 5].

In the present study, the association between EBV serostatus and serum GADA levels has been investigated. Individuals were classified as EBV+ when both VCA IgG and EBNA IgG were detected, reflecting a past infection as VCA IgG appears generally two weeks post-infection and EBNA IgG several weeks or months later. Samples with isolated VCA or EBNA IgG were excluded, since these atypical profiles, although seen in practice in patients with past EBV infection, may reflect recent infection (isolated VCA IgG) or a false-positive results (low positive isolated VCA or EBNA IgG) [12, 13].

The global EBV seroprevalence of our cohort was 57.31%. EBV+ individuals were significantly older than EBV- individuals (median 15 vs. 12.5, *p* = 0.001), with a significant increase of seroprevalence (11%) for each year of age. Our results are consistent with expectations as it is well known that EBV seroprevalence increases with age, and that EBV seroprevalence in France is ranging from ~ 40% (1–4 years old) to ~ 80% (19–24 years old) [14].

The overall F-test indicates that the regression model was statistically significant. The R² value (10.1%) is consistent with the inherent biological variability of antibody levels, as commonly observed in complex multifactorial systems [15]. Age and sex both significantly influence serum GADA levels. In our cohort, women had approximately two-fold higher mean serum GADA levels than men, which is in agreement with results of previous studies showing that both serum GADA levels and GADA seroprevalence are higher in women [16–18].

The main finding of this study is that EBV+ individuals had, on average, 48% lower serum GADA levels than EBV-seronegative individuals after adjustment for age and sex. As EBV+ individuals were older than EBV- individuals, and increasing age was associated with higher GADA levels, this likely explains why the difference between the two groups was not significant in the unadjusted analysis but became significant after adjustment.

To the best of our knowledge, this inverse relationship between EBV seropositivity and serum GADA levels has not been described yet. Nevertheless, it is reminiscent of an inverse relationship between EBV infection and autoantibody production that was previously reported in an ex-vivo study. It has been shown that among B cells from EBV-infected patients, EBV+ memory B cells produce fewer self-reactive antibodies than EBV- memory B cells, notably insulin-reactive antibodies [19].

Taken together, these observations suggest that EBV may downregulate the production of autoantibodies, such as GADA, involved in pancreatic islet autoimmunity, possibly through its immunomodulatory properties. However, we cannot rule out a reverse causal relationship in which higher islet autoantibodies levels (exemplified here by GADA) may protect against EBV infection, or the role of confounding factors (such as genetic factors, immune maturation, or timing of exposure), which would predispose individuals to both EBV susceptibility (or infection) and lower GADA levels.

Further studies are needed to determine whether there is a relationship between EBV infection, islet-specific autoantibodies, HLA genotype and clinical outcome of autoimmunity.

It has been shown that higher EBNA IgG levels are associated with autoimmunity [1, 20], and that patient with newly diagnosed T1D have lower VCA IgG levels than healthy controls [4]. However, in the present study we found no association between VCA or EBNA IgG levels and GADA levels.

## Conclusion

The lower GADA levels in EBV+ individuals suggest that there is an inverse relationship between pancreatic islet autoimmunity and EBV serostatus. Further studies are needed to investigate the role of EBV in pancreatic islet autoimmunity and will be directed along this line in our laboratory.

## Data Availability

The data supporting these findings are included in the main manuscript and are available from the corresponding author on reasonable request.
